# The Role of LncRNAs in the Regulation of Radiotherapy Sensitivity in Cervical Cancer

**DOI:** 10.3389/fonc.2022.896840

**Published:** 2022-05-26

**Authors:** Hanqun Zhang, Chunju Fang, Zhiyu Feng, Tingting Xia, Liang Lu, Min Luo, Yanping Chen, Yuncong Liu, Yong Li

**Affiliations:** ^1^ Department of Oncology, Guizhou Provincial People’s Hospital, Guizhou, China; ^2^ Department of Nephrology, Guizhou Provincial People’s Hospital, Guizhou, China

**Keywords:** LncRNA, cervical cancer, radiotherapy, sensitivity, mechanism

## Abstract

Cervical cancer (CC) is one of the three majors gynecological malignancies, which seriously threatens women’s health and life. Radiotherapy (RT) is one of the most common treatments for cervical cancer, which can reduce local recurrence and prolong survival in patients with cervical cancer. However, the resistance of cancer cells to Radiotherapy are the main cause of treatment failure in patients with cervical cancer. Long non-coding RNAs (LncRNAs) are a group of non-protein-coding RNAs with a length of more than 200 nucleotides, which play an important role in regulating the biological behavior of cervical cancer. Recent studies have shown that LncRNAs play a key role in regulating the sensitivity of radiotherapy for cervical cancer. In this review, we summarize the structure and function of LncRNAs and the molecular mechanism of radiosensitivity in cervical cancer, list the LncRNAs associated with radiosensitivity in cervical cancer, analyze their potential mechanisms, and discuss the potential clinical application of these LncRNAs in regulating radiosensitivity in cervical cancer.

## Background

Cervical cancer is the most common malignant tumor in the female reproductive system, which seriously threaten women’s health and life (1). The current treatment methods for cervical cancer include surgery, radiotherapy, chemotherapy and immunotherapy (2). However, the 5-year survival rate is still not satisfactory, as most patients with cervical cancer are usually diagnosed at an advanced stage or have distant lymph node metastasis (3). As the main treatment for cervical cancer, radiotherapy is applied in all stages of cervical cancer. However, 44% of patients still experience recurrence, and 35% of recurrent tumors are local and regional (4). The failure of radiotherapy is mainly attributed to radioresistance, which exists in radioresistant cancer cell subgroups (5, 6). Radioresistance is the main obstacle to the complete recovery of cervical cancer patients after receiving a complete treatment regimen (7). Therefore, reducing the radiotherapy resistance of cervical cancer and improving the radiotherapy sensitivity of cervical cancer are urgent problems to be solved by clinicians and researchers.

Long non-coding RNAs (LncRNAs) are a group of nonprotein-coding RNAs with a length of more than 200 nucleotides, which are abnormally expressed in many cancers. LncRNAs play an important role in regulating multiple biological processes such as proliferation, apoptosis, migration, invasion, angiogenesis, abnormal metabolism and immune escape (8–11). LncRNAs also directly regulate the transcription and translation of target genes in various ways, or indirectly regulate the genes upstream or downstream of their target genes (12). In addition, lncRNAs are involved in a variety of cell life activities, such as gene imprinting, gene recombination, chromatin modification and cell cycle regulation (13–17). In recent years, increasing evidence has shown that lncRNAs are closely related to the radiosensitivity of malignant tumors (18, 19) and participate in the regulation of radiosensitivity in esophageal cancer (20), nasopharyngeal cancer (21), colorectal cancer (22), pancreatic cancer (23), gastric cancer (24), liver cancer (25), lung cancer (26) and other tumors. Recently, a certain number of lncRNAs have been shown to play an important role in the regulation of radiotherapy sensitivity in cervical cancer. However, there is no summary of lncRNAs involved in the regulation of radiotherapy sensitivity in cervical cancer. Here, we highlight the structure and function of lncRNAs and the mechanism of radiotherapy resistance in tumor cells. In addition, lncRNAs regulating the radiotherapy sensitivity of cervical cancer are summarized and their mechanism of action in cervical cancer is analysed, providing useful information for the study of lncRNAs regulating the radiotherapy sensitivity of cervical cancer.

## Structure and Function of LncRNAs

In the human genome, more than 90% of the genome can be transcribed (27), which leads to the formation of a complex transcription network, which includes tens of thousands of lncRNAs (28). LncRNAs are over 200 nucleotides in length, polyadenylated, selectively spliced, low in abundance and have a relatively low sequence conservation. LncRNAs mainly exist in mammalian epigenetic systems (29, 30). LncRNAs are mainly located in the nucleus or cytoplasm (31, 32). The secondary structure of lncRNA is known through genome-wide studies, which have revealed them to have a hierarchical structure containing subdomains of modular RNA secondary motifs (33, 34). A triple helix is formed at the 3’ end to protect it from RNase degradation (35). The lncRNA structure is more complex than that of mRNA but simpler than that of rRNA (36, 37). Through the determination of the secondary structure of lncRNAs, lncRNAs can be divided into the following groups: 1. highly structured RNAs with subdomains and complex structural motifs; 2. loose RNA with multiple stem rings, but lacking a hierarchical domain and complex motifs; 3. unstructured and disordered RNA, lacking secondary structure (29). Currently, there are few studies on the tertiary structure of lncRNA, and the specific structure is not very clear. Uroda et al. used the chemical detection method to study lncRNA MEG3 and found that its tertiary structure was consistent with the secondary structure (38). Borodavka et al. found that the tertiary structure of lncRNA HOTAIR was more compact than mRNA transcripts but not as compact as ribosomes (39).

LncRNAs are more than 200 nucleotides in length, and their nucleotide sequence structure is similar to that of mRNAs, but lncRNAs lack a standard open reading frame (ORF), and thus they do not encode functional proteins (40, 41). Although lncRNAs do not encode proteins, they still have many biological functions in human diseases. With further research, the functions of lncRNAs in human diseases have been preliminarily clarified. The main biological functions include the following: (1) interfering with the transcription of target genes into mRNA; (2) regulating chromatin remodelling and histone modification; (3) interfering with the mRNA splicing sequence; (4) formation of interfering RNA that binds to target mRNA and leads to its degradation; (5) change protein structure and function and regulating protein activity; (6) acting as miRNA to regulate protein activity; (7) changing intracellular protein localization; and (8) production of small RNA (42).

## The Mechanism of Radioresistance in Malignant Tumors

**Figure 1:**
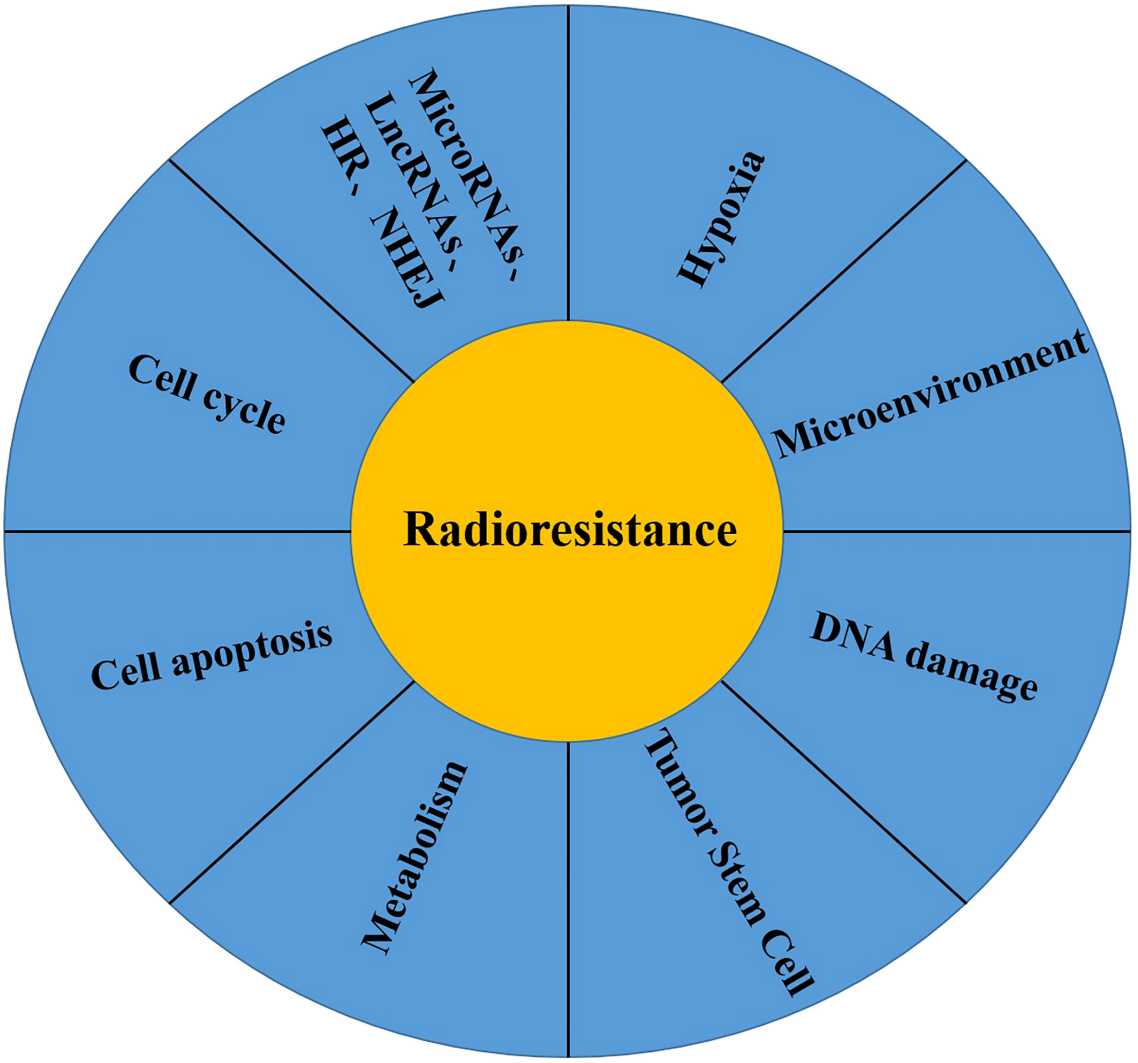
Mechanisms of radiotherapy resistance of malignant tumor cells: (1) Tumor Hypoxia; (2) Tumor Microenvironment; (3) DNA damage repair; (4) Tumor stem cell; (5) Tumor Metabolism; (6) Cell Apoptosis; (7) Cell Cycle; (8) Others: MicroRNA, LncRNAs, HR, NHEJ.

Cancer is a serious threat to human health (43), and radiotherapy is one of the main treatment methods for cancer. Approximately 60% of cancer patients require radiotherapy (44), and radiotherapy can be combined with surgery, chemotherapy and immunotherapy to improve the surgical resection rate and cure rate of cancer patients (45). Despite the improvement of radiotherapy techniques and methods, tumor recurrence or metastasis eventually results due to the inherent or acquired radioresistance of cancer cells (46, 47). Radioresistance is a complex biological process, and the mechanism of radioresistance is not clear at present. The possible mechanisms are described as follows (**Figure 1**): (1) Tumor hypoxia: The significant increase in HIF1α activity in tumor hypoxia induces radioresistance (48). In addition, the reduction in free radical oxidative stress induced by radiotherapy under hypoxic condition also leads to radioresistance (49). (2) Tumor microenvironment: In addition to tumor cells, the tumor microenvironment also includes the nontumor cell matrix, blood vessels, peripheral cells, immune cells and tumor stem cells. Radiotherapy can induce vascular injury and lead to tumor hypoxia, which triggers an immune response by increasing the production of cytokines/chemokines that induce the recruitment of immune cells (50, 51). Local inhibitory immune cells (TAMs,MDSCs and Tregs) increase after radiotherapy, resulting in decreased radiosensitivity (52–54). (3) DNA damage repair: After radiotherapy, DNA double-strand or single-strand breaks can be caused, leading to chromosome aberration or cell death (55). Ataxia telangiectasia mutation (ATM) dependent DNA damage response (56), nonhomologous terminal junction (57), single-strand broken repair (58) and homologous recombination pathways synergistically promote the increased expression of repair genes such as those of the DNA damage repair pathway in tumors (59), which is one of the main causes of radioresistance. (4) Tumor stem cells: Tumor stem cells have the ability to self-renew, differentiate into other tumors and resist tumor treatment, which is one of the main causes of tumor recurrence and metastasis. The number of tumor stem cells and the inherent radioresistance of tumor stem cells are related to the resistance to radiotherapy (60). In addition, non-tumor stem cells can be induced to develop into tumor stem cells after radiotherapy (61). (5) Metabolic changes: Cancer is closely related to metabolic disorders (62), and metabolic reprogramming is also considered as one of the markers of cancer (63). In addition, metabolic changes are related to radiotherapy resistance (64), glucose metabolism can affect radiotherapy sensitivity (65), and regulating mitochondrial function can induce a radiotherapy resistance response (66). (6) Cell cycle, apoptosis and other signalling pathways: In the study of double-strand break repair, HR (homologous recombination) and NHEJ (nonhomologous end-joining) were the two pathways first discovered, HR is triggered in the late S and G2/M stages (67), and NHEJ is triggered in the G0/G1 and G2/M phases (68), and these two pathways are closely related to radiosensitivity (69). DNA is damaged after radiotherapy, leading to apoptosis, and avoidance of apoptosis is one of the main causes of radioresistance in tumor cells (70). In contrast, it participates in the occurrence of radioresistance by regulating the expression of apoptotic proteins (71). (7) Others: In addition to the mechanism mentioned above, radioresistance of tumor cells is also related to tumor heterogeneity (49), microRNAs (72) and lncRNAs (73).

## Mechanism of Radiotherapy for Cervical Cancer

Cervical cancer is one of the main causes of death in females worldwide (74), and radiotherapy is one of the main treatment methods for cervical cancer which is not only applicable in the early stages, but can also be used for advanced and metastatic lesions (75). The standard radiotherapy regimen for cervical cancer is external pelvic irradiation and brachytherapy (76). The main mechanism by which radiotherapy kills tumor cells is the induction of single - and double-stranded DNA breaks. One way to kill tumor cells is by directly damaging their DNA with radiation. The other way is that radiation causes water decomposition and forms free radicals, leading to the indirect death of tumor cells (77). In the treatment of cervical cancer, approximately 80% of patients with cervical cancer require radiotherapy (78). However, radioresistance may occur when irradiated cancer cells adopt substitution mechanisms to promote their survival, proliferation, invasion and escape from cell death in external irradiation or brachytherapy (79). In addition, due to local tumor hypoxia and the existence of tumor stem cells (49, 60), cervical cancer cells can also be resistant to radiotherapy. Therefore, radioresistance is still the main cause of the decline in overall survival and disease-free survival in cervical cancer (80), and is also the main cause of local recurrence and metastasis (4).

## The Role of LncRNAs in Regulating Radiosensitivity of Cervical Cancer

**Figure 2:**
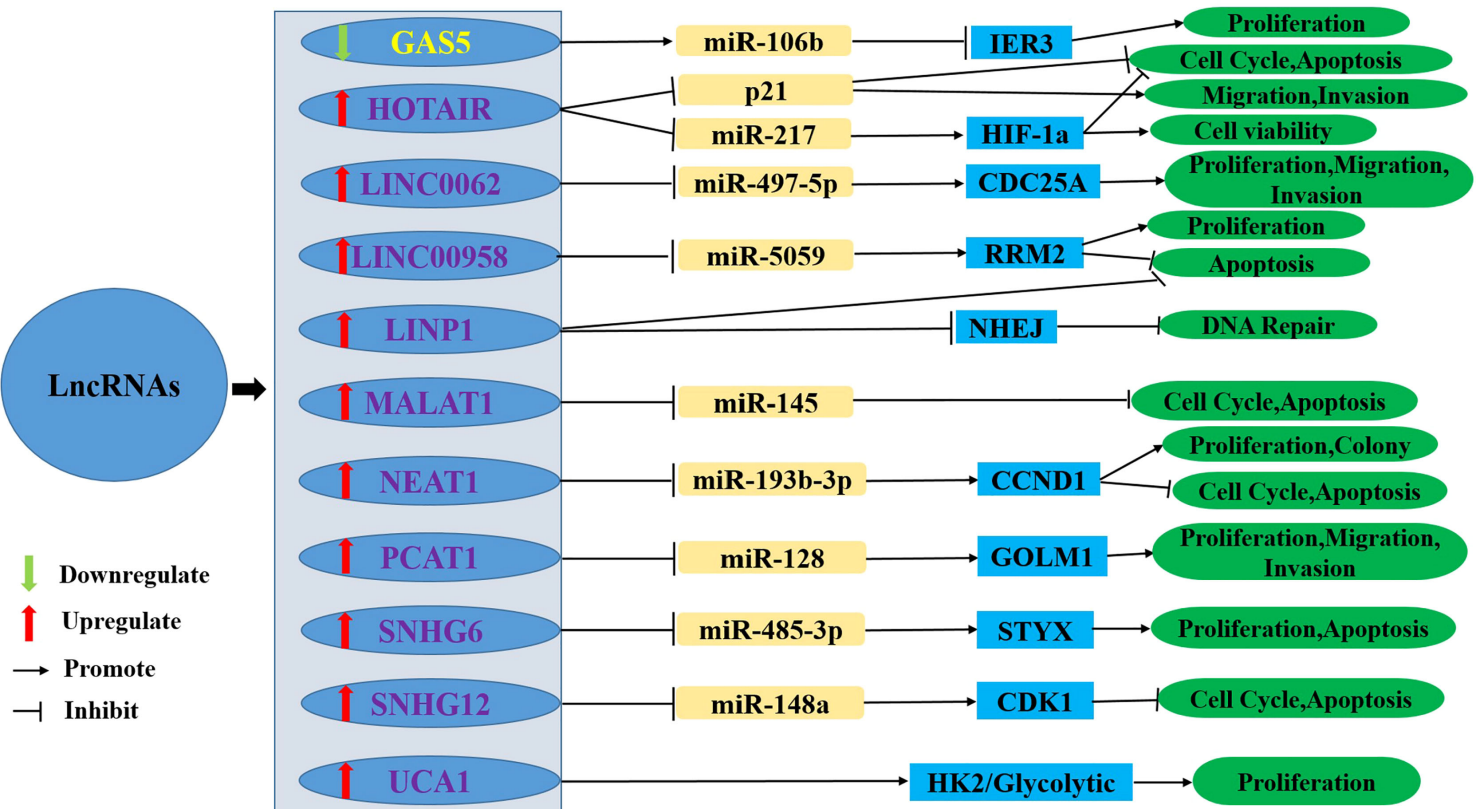
The upregulation or downregulation of LncRNAs leads to radioresistance of cervical cancer through different pathways. LncRNA, Long non-coding RNA; HIF1-α, Hypoxia-Inducible Factor 1-α; NHEJ, nonhomologous end-joinin; GAS5, Growth Arrest Special 5; IER3, immediate early response 3; HOTAIR, HOX Transcript Antisense Intergenic RNA; LINC00662, Long Intergenic Noncoding RNA00662; CDC25A, cell division cycle 25 A; LINC00958, Long Intergenic Noncoding RNA00958; RRM2, ribonucleotide reductase subunit M2; LINP1, LncRNA in non-homologous end joining (NHEJ) Pathway 1; MALAT1, Metastasis-associated lung adenocarcinoma transcript 1; NEAT1, Nuclear-enriched Transcript 1; CCND1, cyclin D1; PCAT1, Prostate cancer- Associated Transcript 1; GOLM1, Golgi membrane protein 1; SNHG, Small nucleolar RNA host gene; CDK1, cyclin-dependent kinase 1; UCA1, Urothelial cancer associated 1; HK2, hexokinase 2.

With the development of scientific and technological means, an increasing number of clinical workers and researchers are paying attention to the correlation between lncRNAs and cervical cancer radioresistance. The abnormal expression of some lncRNAs regulating cervical cancer radioresistance has been studied, and the relevant mechanism has been preliminarily clarified at the molecular level. An increasing number of studies have reported that some lncRNAs can enhance radioresistance, while some lncRNAs can reduce radioresistance. This review systematically summarised the lncRNAs involved in the regulation of radioresistance in cervical cancer, reviews the recent studies on the regulation of radioresistance to cervical cancer by lncRNAs, and described their possible mechanisms (**Figure 2** and **Table 1**).

**Table 1:** Functional characterization of LncRNAs in Cervical cancer.

LncRNAs	Expression	Function	Mechanism	Role	Reference
GAS5	Downregulation	Proliferation	miR-106b/IER3	Anti-oncogene	(81)
HOTAIR	Upregulation	Cell cycle, apoptosis, cell viability, migration, invasion	P21/miR-217/HIF-1a	Oncogene	(82, 83)
LINC00662	Upregulation	Proliferation, migration, invasioninvasion	miR-497-5p/CDC25A	Oncogene	(84)
LINC00958	Upregulation	Proliferation, apoptosis	miR5059/RRM2	Oncogene	(85)
LINP1	Upregulation	Proliferation, DNA repair	NHEJ	Oncogene	(86)
MALAT1	Upregulation	Cell cycle, apoptosis	miR-145	Oncogene	(87)
NEAT1	Upregulation	Proliferation, apoptosis,Cell cycle, colony	miR-193b-3p/CCND1	Oncogene	(88)
PCAT1	Upregulation	Proliferation, migration, invasion	miR-128/GOLM1	Oncogene	(89)
SNHG6SNHG12UCA1	Upregulation Upregulation Upregulation	Proliferation, apoptosisCell cycle, apoptosisProliferation	miR-485-3p/STYXmiR-148a/CDK1HK2/Glycolytic	Oncogene Oncogene Oncogene	(90) (91) (92, 93)

LncRNA, Long non-coding RNA; HIF1-α, Hypoxia-Inducible Factor 1-α; NHEJ, nonhomologous end-joinin; GAS5, Growth Arrest Special 5; IER3, immediate early response 3; HOTAIR, HOX Transcript Antisense Intergenic RNA; LINC00662, Long Intergenic Noncoding RNA00662; CDC25A, cell division cycle 25 A; LINC00958, Long Intergenic Noncoding RNA00958; RRM2, ribonucleotide reductase subunit M2; LINP1, LncRNA in non-homologous end joining (NHEJ) Pathway 1; MALAT1, Metastasis-associated lung adenocarcinoma transcript 1; NEAT1, Nuclear-enriched Transcript 1; CCND1, cyclin D1; PCAT1, Prostate cancer- Associated Transcript 1; GOLM1, Golgi membrane protein 1; SNHG, Small nucleolar RNA host gene; CDK1, cyclin-dependent kinase 1; UCA1, Urothelial cancer associated 1;HK2, hexokinase 2.

### Growth Arrest Special 5 (GAS5)

GAS5 is a tumor suppressor whose expression level increases during growth stagnation (94). There are approximately 630 nucleotides in GAS5, consisting of 5’ -terminal oligopyrimidine RNA composed of 12 nonconserved exons, located on chromosome 1q25 (95, 96). Studies have shown that the expression levels of GAS5 is positively correlated with the apoptosis of cervical cancer cells and the sensitivity of chemotherapy drugs, and negatively correlated with the proliferation and metastasis of cervical cancer cells (97, 98). Recent studies have shown that GAS5 is positively correlated with radiotherapy sensitivity in cervical cancer. Gao et al. (81) used qRT-PCR to assess 11 radio-sensitive cervical cancer tissues and 9 radio-resistant cervical cancer tissues, and the results showed that GAS5 expression in the radioresistant tissues was significantly reduced compared with radiosensitive tissues. In HeLa cells, overexpression of GAS5 reduced the cell survival score and promoted radiotherapy sensitivity, while in ME180 cells, GAS5 knockdown increased the cell survival score and led to radiotherapy resistance. Animal experiments showed that when mice were irradiated with 6 MV X-ray 16Gy/4F, the tumor size of the GAS5-overexpressing group was significantly reduced, while that of the GAS5 knockdown group was significantly increased. Further mechanistic studies showed that overexpression of GAS5 resulted in upregulation of immediate early response 3(IER3) and downregulation of miR-106b, thus increasing radiological sensitivity. However, upregulation of miR-106b or downregulation of IER3 could reverse this effect. The experimental results showed that GAS5 enhanced the sensitivity of cervical cancer cells to radiotherapy by inhibiting miR-106b and upregulating IER3.

### HOX Transcript Antisense Intergenic RNA (HOTAIR)

HOTAIR is transcribed from the antisense strand of the HOXC gene cluster with 2158 nucleotides and 6 exons located between HoxC11 and HoxC12 on the chromosome 12Q13.13 (99). Studies have fully proven that HOTAIR is highly expressed in cervical cancer tissues and cells, and is related to tumor proliferation and metastasis (82, 83). Moreover, HOTAIR can modulate the radiotherapy sensitivity of cervical cancer. Li et al. (100), when studying the radiotherapy sensitivity of HOTAIR to cervical cancer, found that downregulated expression of HOTAIR significantly increased the radiosensitivity of HeLa cells and induced G1 phase arrest and apoptosis, but this effect could be suppressed by downregulation of P21, and the upregulation of P21 made these cells regain their sensitivity to radiation. Overexpression of HOTAIR increased the resistance of C33A cells to radiation and promoted C33A cells to enter S phase, but this effect could be reversed by overexpression of P21. The results showed that HOTAIR regulates the sensitivity of cervical cancer cells by acting on P21. Another study showed that the expression of HOTAIR in HeLa and C33A cells was significantly higher than that in normal cervical cells, but after irradiation of HeLa and C33A cells, the expression of HOTAIR was significantly decreased in a time-dependent manner, which inhibited the viability of cervical cancer cells and promoted apoptosis of cervical cancer cells. However, this effect could be eliminated by the overexpression of HOTAIR. Further mechanistic studies showed that HOTAIR regulates the radiosensitivity of cervical cancer cells and is related to HIF-1α expression. After irradiation, HeLa and C33A cells overexpressing HOTAIR increased HIF-1α expression, leading to radioresistance and promoting tumor growth, but this effect could be neutralized by miR-217 mimics. Under hypoxic condition, the inhibition of miR-217 expression by HOTAIR could be upregulated. Thus, HIF-1α expression was promoted to increase the radioresistance of cervical cancer cells. In contrast, knockdown of HOTAIR increased the radiosensitivity of cervical cancer cells by increasing the expression of miR-217 and decreasing the expression of HIF-1α (101).

### Long Intergenic Noncoding RNA00662 (LINC00662)

LINC00662 is located on chromosome 19q11 and is 2085 bp long. LINC00662 is upregulated in a variety of malignant tumors, and the upregulated expression of LINC00662 is also closely related to the poor prognosis, radioresistance and chemoresistance of cancer patients (102, 103). The expression of LINC00662 was shown to be significantly upregulated in cervical cancer tissues and cells, and the high expression of LINC00662 resulted in reduced overall survival and relapse-free survival, and was associated with FIGO stage and lymph node metastasis. Overexpression of LINC00662 significantly increased the proliferation, migration and invasion of these cells, and increased the radioresistance of C33A cells. LINC00662 inhibited the proliferation, migration and invasion of Caski cells, and increased the radiosensitivity of Caski cells. In C33A cells, overexpression of LINC00662 resulted in significant downregulation of miR-497-5p, while the overexpression of miR-497-5p partially reversed the promotion by overexpressed LINC00662 of proliferation, metastasis and radioresistance of C33A cells. In Caski cells the effect was reversed. Overexpression of LINC00662 or miR-497-5p inhibition resulted in the significantly increased cell division cycle 25 A(CDC25A) expression, while knockdown of LINC00662 or overexpression of miR-497-5p resulted in decreased CDC25A expression. The results showed that LINC00662 promotes the proliferation, migration, invasion and radioresistance of cervical cancer cells through the LINC00662/miR-497-5p/CDC25A axis (84).

### Long Intergenic Noncoding RNA00958 (LINC00958)

The expression of LINC00958 in cervical cancer tissue was higher than that in normal tissue, and the high expression of LINC00958 was negatively correlated with the overall survival of cervical cancer. In addition, in HeLa cells with LINC00958 knockdown, sh-LINC00958 inhibited the proliferation of HeLa cells, increased cell apoptosis after 6Gy X-ray irradiation, and reduced tumor volume and tumor weight in mice. Moreover, sh-LINC00958 can upregulate the expression of miR-5095 and inhibit ribonucleotide reductase subunit M2 (RRM2). Studies have shown that sh-LINC00958 can increase the radiosensitivity of cervical cancer. Further bioinformatics analysis and dual luciferase assays confirmed that LINC00958 can regulate miR-5095, which can regulate RRM2 by binding to its 3’-UTR. In HeLa cells transfected with sh-RRM2, after 6 Gy X-ray irradiation, sh-RRM2 inhibited cell proliferation and clonal formation, and increased cell apoptosis, while overexpressed RRM2 had the opposite effect. Upregulation of miR-509 can inhibit cell proliferation, induce apoptosis, and downregulate the expression of RRM2. After treatment with miR-509 inhibitor, this effect was reversed. Therefore, the above results suggest that LINC00958 silencing promotes the sensitivity of cervical cancer cells to radiotherapy by regulating the miR-5095/RRM2 axis (85).

### LncRNA in Nonhomologous End Joining (NHEJ) Pathway 1 (LINP1)

The LINP1 gene is located on chromosome 10:6737382-6739026 and is 917bp in length. It is an important component of the NHEJ synaptic complex (104). LINP1 is highly expressed in most malignant tumor tissues and cells, promotes tumor progression, inhibits apoptosis, and promotes resistance to chemotherapy and endocrine drugs (105–107). In addition, LINP1 can also promote the repair of DNA double-strand damage to breast cancer cells after radiotherapy, leading to radiotherapy resistance (104). LINP1 was shown to be highly expressed in cervical cancer tissues and HeLaS3 cells. The RNA- fluorescence *in situ* hybridization technique showed that LINP1 was mainly distributed in the cytoplasm. After radiation treatment, LINP1 was translocated to the nucleus, and the expression of LINP1 increased 24 hours after radiation, but this effect could be blocked by EGFR inhibitors. It is thought that the expression of LINP1 may be regulated by the EGFR pathway in cervical cancer cells. In addition, knockdown LINP1 can increase radiosensitivity, mainly by promoting apoptosis by increasing cleaved caspase3 and PARP expression levels; on the other hand, DNA damage repair was inhibited by increasing γ-H2AX expression. These results suggest that LINP1 increases radioresistance in cervical cancer cells by inhibiting apoptosis and promoting DSB repair through the NHEJ pathway (86). Therefore, LINP1 may serve as a prognostic marker and a potential therapeutic target for cervical cancer treatment.

### Metastasis-Associated Lung Adenocarcinoma Transcript 1 (MALAT1)

MALAT1 is transcribed by RNA polymerase II. Its promoter has an accessible open chromatin structure, and this gene is encoded on the human chromosome 11q13 with a length of ~8.7 knt and is highly evolutionarily conserved (31). MALAT1 was initially found to be associated with lung cancer, but recently MALAT1 has been found to be abnormally expressed in most cancers, working as a decoy for splicing factors leading to splicing malfunctioning (108). In cervical cancer, Lu et al. performed qRT-PCR assessment on 21 radio-sensitive HR-HPV + (high-risk human papillomavirus+) and 29 radio-resistant cervical cancer tissues, and the results showed that the expression of MALAT1 in radiation-resistant cervical cancer tissues was higher than that in radiation-sensitive cervical cancer tissues. In addition, the expression of miR-145 was negatively correlated with MALAT1 expression in cervical cancer tissues. In MALAT1-knockout HR-HPV-16+ CaSki and HR-HPV-18+ HeLa cells, irradiation reduced the cell formation rate and the proportion of G1 phase cells, but increased the proportion of G2/M phase cells and apoptosis. In addition, the overexpression of miR-145 significantly reduced the rate of CaSki and HeLa cell clonal formation, while the overexpression effect of miR-145 combined with MALAT1 knockout was more significant than that of miR-145 alone. The results showed that MALAT1 regulates the radiosensitivity of HR-HPV+ cervical cancer cells by regulating miR-145 (87).

### Nuclear-Enriched Transcript 1 (NEAT1)

NEAT1 is located on 11q13.1 in human chromosomes, and consists of two subtypes, NEAT1-1 and NEAT1-2. These two subtypes share a common promoter and initiation site. However, the termination sites are different. In addition, between the two subtypes, the 3’end of the NEAT1-1 transcript is typically polyadenylated. However, the 3’ end of the NEAT1-2 transcript is not-polyadenylated (109). In recent years, the role of NEAT1 in malignant tumors has been more reported. In cervical cancer, NEAT1 is not only related to the growth, metastasis and prognosis of cervical cancer, but also can regulate sensitivity to chemotherapy drugs (110–112). To understand whether NEAT1 regulates the radiosensitivity of cervical cancer, Han et al. used qRT-PCR to detect the expression of NEAT1 in cervical cancer tissues and radioresistant cervical cancer cells (HeLa-R and SiHa-R). NEAT1 expression was significantly increased in cervical cancer tissues and radioresistant cervical cancer cells. In addition, high NEAT1 expression was associated with prognosis, lymph node metastasis, tissue differentiation and TNM score in tumor patients. To further confirm the correlation between NEAT1 and radiosensitivity of cervical cancer, they transfected HeLa and SiHa cells to overexpress NEAT1. After the transfection of HeLa-R and SiHa-R cells to knockdown NEAT1, the proliferation in the overexpression group increased compared with that in the negative control cells after different doses of irradiation. Compared with the negative control group, the cell proliferation of the knockdown group decreased. It was thought that NEAT1 expression increased radiation resistance, and that NEAT1 knockdown increased radiation sensitivity. To understand the mechanism by which NEAT1 regulates the radiosensitivity of cervical cancer, Han et al. demonstrated that NEAT1 was correlated with miR-193b-3p through bioinformatics analysis, and therefore they assessed overexpressed NEAT1 and knocked down NEAT1 in cells. In NEAT1-overexpressing cells, the expression of miR-193b-3p decreased. However, miR-193b-3p expression was increased in knockdown NEAT1 cells. It is considered that NEAT1 negatively regulates the expression of miR-193b-3p to affect the radiosensitivity of cervical cancer. Further studies showed that cyclin D1 (CCND1) is one of the targets of miR-193b-3p. By upregulating miR-193b-3 expression and downregulating CCND1 expression, NEAT1 silencing can reduce the cell survival rate and clone formation, and increase G0/G1 phase cell cycle arrest and apoptosis, thus improving the radiosensitivity of cervical cancer cells. This may indicate that NEAT1 knockdown regulates the radiosensitivity of cervical cancer through the NEAT1/miR-193b-3p/CCND1 signalling pathway (88).

### Prostate Cancer-Associated Transcript 1 (PCAT1)

PCAT1 is a polyadenylated lncRNA containing 1.9 kb and two exons. Among Chr8q24 gene located approximately 725 kb upstream of the MYC oncogene, PCAT1 was initially found in prostate cancer and is involved in the occurrence and development of prostate cancer (113). Subsequently, the abnormal expression of PCAT1 was found to be related to the occurrence and development of a variety of malignant tumors, and can lead to chemotherapy resistance (114, 115). In addition, PCAT1 can also regulate the radiosensitivity of malignant tumors (116). The expression levels of PCAT1 were positively correlated with FIGO stage, lymph node metastasis and the depth of cervical tumor invasion. *In vitro* experiments showed that knocking down PCAT1 could improve the radiosensitivity by inhibiting the proliferation, migration and invasion of cervical cancer. Similarly, in animal experiments, knocking down PCAT1 inhibited tumor growth, reduced tumor volume and weight, and significantly enhanced the antitumor effect of radiation compared with the NC group. Further mechanistic studies showed that miR-128 is a downstream target of PCAT1 and is negatively regulated by PCAT1. Knockdown of PCAT1 leads to upregulation of miR-128, which enhances the radiosensitivity of cervical cancer cells to proliferation, migration, and invasion. Golgi membrane protein 1(GOLM1) is a downstream target of miR-128 and is negatively regulated by miR-128. Both upregulation of GOLM1 and downregulation of miR-128 can reverse the enhanced radiosensitivity effect of PCAT1 gene knockout on cervical cancer cells, thus promoting the proliferation, migration and invasion of cervical cancer cells (89).

### Small Nucleolar RNA Host Gene (SNHG)

Small nucleolar RNA host genes (SNHGs) are long non-coding RNAs that have been reported to be expressed in a variety of cancers. SNHG1, SNHG3, SNHG5, SNHG6, SNHG7, SNHG12, SNHG15, SNHG16, and SNHG20 can induce proliferation, cell cycle progression, invasion and metastasis of cancer cells, possibly making SNHGs effective biomarker for cancer progression and invasion (117). SNHG6 is highly expressed in cervical cancer tissues and cell lines, especially in SiHa, HeLa and CaSki cells. Knockdown of SNHG6 can inhibit tumor cell growth and promote apoptosis, suggesting that SNHG6 plays a role in cervical cancer by promoting tumor cell growth and enhancing radiation resistance. Bioinformatics analysis and MS2-RIP analysis were used to prove that SNHG6 was cross-linked with miR-485-3p, and that STYX was the downstream target of miR-485-3p. SNHG6 is negatively correlated with miR-485-3p. Downregulation of SNHG6 or overexpression of miR-485-3p can reduce the expression level of STYX, while knockdown of miR-485-3p or overexpression of STYX can eliminate the effect of SNHG6 silencing on the growth of cervical cancer cells. Studies have shown that SNHG6/miR-485-3p/STYX axis contributes to the growth and radiation resistance of cervical cancer cells, thus promoting the progression of cervical cancer (90). Similarly, SNHG12 was overexpressed in both cervical cancer tissues and cells, and was positively correlated with tumor size and TNM stage. Interestingly, however, the expression of SNHG12 in tumor tissues and cells after radiotherapy was significantly lower than that before treatment, suggesting that SNHG12 may be related to the radiotherapy sensitivity of cervical cancer. Cell and animal experiments have shown that SNHG12 knockdown can inhibit tumor growth by increasing apoptosis and cell cycle tissue, thus improving radiosensitivity. Further mechanistic research, through bioinformatics analysis, shown that SNHG12regulates the expression of cyclin-dependent kinase 1(CDK1) through the adsorption of miR-148a to increase the radiosensitivity of cervical cancer. miR-148a is expressed at low levels in cervical cancer tissues and cell lines, and the low expression of miR-148a is positively correlated with the patient’s tumor size and TNM stage, which indicates that SNHG12 is negatively correlated with miR-148a a. Knockdown of SNHG12 plays a role in cervical cancer by increasing the expression of miR-148a, which can be reversed by miR-148a inhibitors. CDK1 is overexpressed in cervical cancer cell lines. In addition, SNHG12 overexpression can promote the expression of CDK1, but this effect can be reversed by miR-148a overexpression. However, miR-148a inhibitor and CDK1 overexpression can reverse the effects of SNHG12 gene knockout on the radiosensitivity of cervical cancer cells (91). These results indicate that the lncRNA SNHG12 regulates radiosensitivity of cervical cancer through the SNHG12/miR-148a/CDK1 regulatory axis and provides a new therapeutic target for the radiosensitivity of cervical cancer.

### Urothelial Cancer Associated 1 (UCA1)

The UCA1 gene contains three exons and two introns and is located on human chromosome 19p13 (118). It was first discovered in studies of bladder cancer (119), has been reported in a variety of malignancies and is involved in radiotherapy resistance and chemotherapy resistance of malignancies (120–122). The expression of UCA1 in cervical cancer tissues is higher than that in normal tissues, and similarly, UCA1 expression in cervical cancer cell lines (HeLa, SiHa, C33A, and CaSKi) is also higher than that in normal cells (92). Fan et al. (93) irradiated HeLa and SiHa cells with 76 Gy X ray to establish HeLa and SiHa radiation resistant cells (HeLa-IRR and SIha-IRR). In HeLa-IRR and SiHa-IRR cells, the expression of UCA1 was significantly higher than that in HeLa and SiHa cells. Upregulation of UCA1 expression is considered to lead to radioresistance. Furthermore, HeLa-IRR and SiHa-IRR cells can increase glycolysis by increasing Hexokinase 2 (HK2), PKM, HIF-1α and GLUT-1 expression levels, and increase radiation resistance of cervical cancer cells. Inhibition of glycolysis increased the radiosensitivity of HeLa-IRR and SiHa-IRR cells. In addition, UCA1 gene knockout inhibited glucose consumption and lactic acid production in SiHa IRR and HeLa IRR cells, and decreased HK2 protein expression levels but did not significantly affect the expression of HIF-1α, GLUT-1, GLUT-4 or PKM. In contrast, overexpression of UCA1 in SiHa and HeLa cells increased glucose uptake and lactate production, as well as HK2 protein expression levels, and had no significant effect on the expression of HIF-1α, GLUT-1, GLUT-4 or PKM. These results suggest that UCA1 may play an important role in regulating radiation resistance through HK2/glycolysis pathway, providing a new potential target for improving radiotherapy for cervical cancer.

## Conclusion and Outlook

Cervical cancer is the fourth most common malignant tumor leading to death in females. Radiotherapy is one of the main treatment methods for cervical cancer, especially for intermediate and advanced cervical cancer, but radiation resistance is the main cause of local recurrence and metastasis of cervical cancer. Although researchers and clinicians have been studying the sensitivity of cervical cancer for many years, the exact mechanism that regulates the radiosensitivity of cervical cancer is still not clear. In this paper, the role and mechanism of lncRNAs in radiotherapy of cervical cancer were summarized and analysed. The structure and function of lncRNAs, the molecular mechanism of radiotherapy resistance in cervical cancer, and the relationship between lncRNAs and radiotherapy resistance in cervical cancer were systematically reviewed. Through analysis, we have learned that lncRNA mainly regulate the radiosensitivity of cervical cancer by mediating DNA damage, regulating the cell cycle, apoptosis, and glycolysis, and targeting miRNAs. Therefore, the regulation of radiation resistance by lncRNAs in cervical cancer has become an important direction in the study of radiation sensitivity in malignant tumors. However, there are no clear guidelines based on lncRNA expression levels to distinguish radiation-sensitive cervical cancer patients from radiation-resistant cervical cancer patients during clinical treatment implementation. However, we believe that with the development of detection and analysis technology, we can predict the treatment effect and prognosis of cancer patients by monitoring related signalling pathways to carry out more effective and precise treatment and individualized treatment, and improve the effect of radiotherapy for cervical cancer.

At present, although several lncRNAs have been confirmed to be related to the radiosensitivity of cervical cancer, there are still many problems to be solved. First, tens of thousands of lncRNAs have been confirmed to be related to human tumor, but only a few lncRNAs are related to the radiosensitivity of cervical cancer. Therefore, we need to discover new lncRNAs and clarify their exact mechanism of action in cervical cancer to obtain more candidate target genes for cervical cancer radiosensitivity. Second, although LncRNAs have rapidly attracted great attention in malignant tumor research in recent years, basic and clinical research are still needed to verify the reliability of LncRNAs and conduct in-depth studies on their molecular mechanisms for clinical application, as there are no clear lncRNA-based guidelines for clinical application to distinguish cervical cancer patients who are sensitive and insensitive to radiotherapy. Finally, we need to adopt advanced, stable and rapid detection technology to conduct rapid and accurate detection of lncRNAs in clinical practice to generate the test results required for clinical examination and treatment, and facilitate clinical prediction of treatment effect and evaluation of treatment prognosis.

## Author Contributions

HZ, CF, and ZF designed the research. HZ drafted the manuscript. CF, ZF, and TX improved the structure of this manuscript and completed the diagrams. YCL, LL,YL, and ML discussed and revised the manuscript. YC, YL, and YCL put forward some constructive suggestions. All authors read and approved the final manuscript.

## Funding

This article was supported by the Science and Technology Foundation Project of Guizhou Health Commission (award number: No. gzwjkj2020-1-031), Guizhou Health Commission (award number: No. gzwjkj2022-027) and Guizhou Science and Technology Planning project (No.: Guizhou Science and Technology Foundation -ZK[2021] General 455).

## Conflict of Interest

The authors declare that the research was conducted in the absence of any commercial or financial relationships that could be construed as a potential conflict of interest.

## Publisher’s Note

All claims expressed in this article are solely those of the authors and do not necessarily represent those of their affiliated organizations, or those of the publisher, the editors and the reviewers. Any product that may be evaluated in this article, or claim that may be made by its manufacturer, is not guaranteed or endorsed by the publisher.

## Abbreviations

CC, Cervical cancer; RT, Radiotherapy; LncRNA, Long non-coding RNA; ORF, open reading frame; HIF1-α, Hypoxia-Inducible Factor 1-α; ATM, Ataxia telangiectasia mutation; HR, Homologous recombination; NHEJ, nonhomologous end-joinin; GAS5, Growth Arrest Special 5; IER3, immediate early response 3; HOTAIR,HOX Transcript Antisense Intergenic RNA; LINC00662, Long Intergenic Noncoding RNA00662; CDC25A, cell division cycle 25 A; LINC00958, Long Intergenic Noncoding RNA00958; RRM2, ribonucleotide reductase subunit M2; LINP1, LncRNA in non-homologous end joining (NHEJ) Pathway 1; EGFR, epidermal growth factor receptor; PARP, poly ADP-ribose polymerase; MALAT1, Metastasis-associated lung adenocarcinoma transcript 1; NEAT1, Nuclear-enriched Transcript 1;CCND1, cyclin D1; PCAT1, Prostate cancer- Associated Transcript 1; GOLM1, Golgi membrane protein 1; SNHG, Small nucleolar RNA host gene; CDK1, cyclin-dependent kinase 1; UCA1, Urothelial cancer associated 1; HK2, hexokinase 2.
